# Activation-induced cytidine deaminase (AID) linking immunity, chronic inflammation, and cancer

**DOI:** 10.1007/s00262-012-1255-z

**Published:** 2012-04-19

**Authors:** Diana Mechtcheriakova, Martin Svoboda, Anastasia Meshcheryakova, Erika Jensen-Jarolim

**Affiliations:** 1 Department of Pathophysiology and Allergy Research, Center for Pathophysiology, Infectiology and Immunology, Medical University of Vienna, Währinger Gürtel 18-20, 1090 Vienna, Austria; 2 Messerli Research Institute of the Medical University of Vienna, Veterinary University of Vienna and University of Vienna, Vienna, Austria

**Keywords:** Activation-induced cytidine deaminase, Gene network, Inflammation, Cancer, Multigene signature approach, AllergoOncology symposium-in-writing

## Abstract

Activation-induced cytidine deaminase (AID) is critically involved in class switch recombination and somatic hypermutation of Ig loci resulting in diversification of antibodies repertoire and production of high-affinity antibodies and as such represents a physiological tool to introduce DNA alterations. These processes take place within germinal centers of secondary lymphoid organs. Under physiological conditions, AID is expressed predominantly in activated B lymphocytes. Because of the mutagenic and recombinogenic potential of AID, its expression and activity is tightly regulated on different levels to minimize the risk of unwanted DNA damage. However, chronic inflammation and, probably, combination of other not-yet-identified factors are able to create a microenvironment sufficient for triggering an aberrant AID expression in B cells and, importantly, in non-B-cell background. Under these circumstances, AID may target also non-Ig genes, including cancer-related genes as oncogenes, tumor suppressor genes, and genomic stability genes, and modulate both genetic and epigenetic information. Despite ongoing progress, the complete understanding of fundamental aspects is still lacking as (1) what are the crucial factors triggering an aberrant AID expression/activity including the impact of Th2-driven inflammation and (2) to what extent may aberrant AID in human non-B cells lead to abnormal cell state associated with an increased rate of genomic alterations as point mutations, small insertions or deletions, and/or recurrent chromosomal translocations during solid tumor development and progression.

## AID under physiological conditions

Adaptive immunity provides defense mechanisms ensuring extreme specificity for foreign antigens with virtually unlimited diversity. B lymphocytes have developed two additional independent steps to further diversify their receptors after antigen collision: somatic hypermutation (SHM) and class switch recombination (CSR). These reactions take place in secondary lymphoid tissues (lymph nodes, tonsils, and spleen) and represent physiological processes that modify variable (V) and constant (C) regions of immunoglobulin (Ig) genes in activated B cells [1]. The discovery of AID [2, 3] (in 1999—mouse ortholog; in 2000—human ortholog) transformed our understanding of basic mechanisms for antibody diversity and the field of immunology as a whole; both SHM and CSR were found to be critically dependent on AID activity.

AID is a member of the APOBEC family of cytidine deaminases, which acts via introduction of single-strand breaks into target DNA through deamination of cytosines into uracils. AID is currently considered as the only B-cell-specific factor required to trigger both SHM and CSR, when DNA breaks are specifically introduced in the variable or switch regions of Ig genes, respectively [4, 5]. Through further processing by DNA-repair enzymes upon recognition of uracil in DNA, this initial single biochemical activity triggers different genetic modifications [6, 7]. As consequence, the production of Igs of various isotypes with high affinity for antigen is achieved. This may especially account for IgE type immunoglobulins exhibiting outstanding affinities for their specific epitopes or allergens. In the germinal center (GC), the AID expression is initiated in early centroblasts, is maximal in full-blown centroblasts, significantly decreases in centrocytes, and is downregulated again in plasma cells. Additionally, the AID-positive cells could be also detected outside the GC; a major fraction of these types of AID-positive cells reside within the subset of interfollicular large B lymphocytes [8, 9].

AID deficiency (as well as defects in the CD40L/CD40 pathway) is among essential causative factors of hyper-IgM (HIGM) syndromes. HIGM are primary immunodeficiencies characterized by the absence of all the isotypes except for elevated IgM [10]. The phenotype observed in HIGM patients and paradoxical observation that AID-deficient subjects often suffer from autoimmune conditions demonstrate the absolute requirement for AID in several crucial steps of B-cell terminal differentiation and suggest an important role of AID for the establishment of both central and peripheral B-cell tolerance. Thus, Meyers et al. [11] identified a novel, previously unsuspected role for AID in the removal of developing autoreactive B cells in humans.

Accumulating evidence suggests another essential role of AID—in two forms of heritable information, namely genetic and epigenetic. The underlying mechanisms behind these two modes of inheritance have so far remained distinct. Given that cytosine deaminases, and particularly AID, have been implicated both in genetic variation of somatic cells and in epigenetic remodeling of germ and pluripotent cells, an audacious hypothesis was proposed by Chahwan et al. [12] that the AID/APOBEC family provide crosstalk between genetic and epigenetic information through cytosine deamination and, moreover, could be important drivers of evolutionary adaptability.

## Multiple levels of AID regulation

**Fig. 1:**
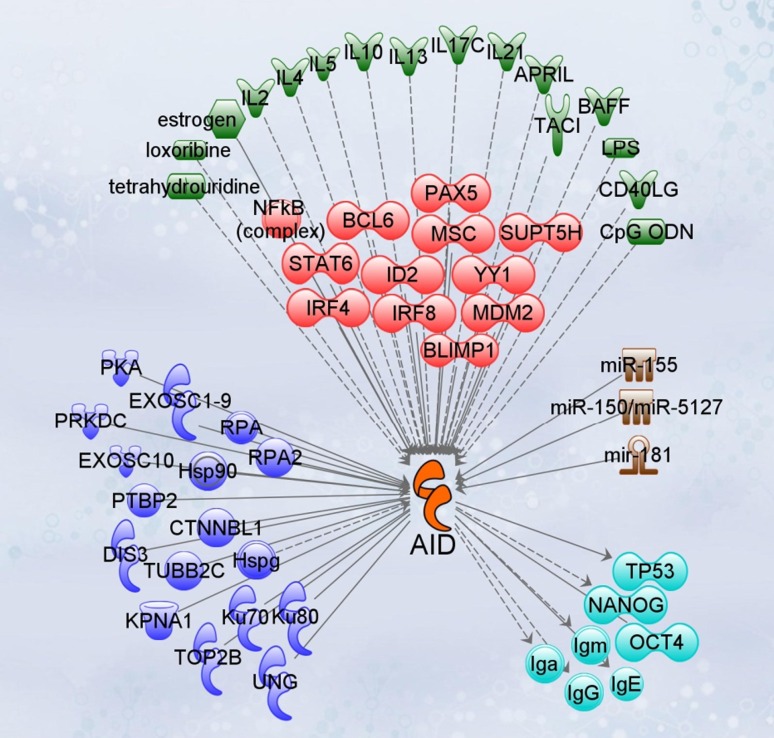
AID-associated gene network. Gene network displaying AID as key gene was created using the Ingenuity Pathway Analysis Software (IPA; http://www.ingenuity.com). Different gene modules are spatially separated for visualization: stimuli controlling AID expression and activity (*green* color code); transcriptional regulators (*red* color code); an additional level of AID regulation is displayed by a group of miRNAs (*brown* color code); direct and indirect AID-interacting molecules associated with functional activity of AID (*blue* color code); molecules modulated by AID activity including various Ig isotypes as consequence of CSR events and examples of affected pluripotency genes as a result of AID-mediated reprogramming and DNA demethylation as well as TP53 being a prominent target of AID as genome-wide mutator (*cyan* color code)

Clearly, such a potent mutagenic and recombinogenic enzyme needs to be tightly regulated on different levels to minimize the risk of unwanted DNA damage (Fig. 1). Indeed, a number of mechanisms restricting AID expression/activity to a distinct cell type, time, and loci were identified. On the transcriptional level, AID is induced in vitro in B cells by Th2 cytokines as IL4 and ligation of CD40; in mice, other inducers have been identified, including LPS [3, 13, 14]. E-protein, NFκB, PAX5, STAT6, and IRF8 transcription factors participate in inducible expression of AID; potential negative regulators, such as IRF4, BLIMP1, ID3, and ID2, may control the stage-specific expression (reviewed by [15]); however, our understanding of the transcriptional regulation of AID gene is not yet complete. In one of the recent publications, HoxC4 was implemented into the AID promoter regulation via binding to a highly conserved HoxC4-Oct site; this site functions in synergy with a conserved binding site for the transcription factors Sp1, Sp3, and NF-κB [16]. Furthermore, AID is known to be regulated on the level of mRNA stability by microRNAs [17–19]. An additional controlling mechanism, which is not yet fully elucidated, is the splicing of AID mRNA. The naturally occurring splice variant lacking exon 4, AID-Δex4 (AY536517), encodes a C-terminally truncated product, which, in contrast to the full-length transcript, is characterized by the complete lack of CSR activity, while showing hyper-SHM activity [20]. It is essential to note that based on our recent data, both AID full-length and AID-Δex4 mRNAs can be detected in chronically inflamed tissues such as nasal polyps and in some normal non-lymphatic tissues as well [21]. Post-translational modification as phosphorylation of threonine 27, threonine 140, and/or serine 38 also regulates AID activity [22–24]. One of the crucial post-translational mechanisms controlling AID functionality including the balance between antibody diversification and off-target mutations and translocations is based on the nucleo-cytoplasmic shuttling: the subcellular localization of AID determines how much AID is in contact with genome. Currently, the following mechanisms influencing human AID subcellular distribution have been characterized: nuclear export [25–27], active nuclear import and cytoplasmic retention [28], and rapid degradation in the nucleus [29]. Finally, even when AID expression/activity is being controlled by multiple mechanisms, one important cellular mechanism, which determines the target specificity of AID, still must function properly. This is yet one of the most interesting questions in AID biology: how does the preferential targeting to Ig loci work and why the specificity of AID is not absolute? It seems that AID recruitment to the particular genes requires a combination of high transcriptional activity of a gene, the presence of high levels of AID targeting hotspots, the presence and activity of protein kinase A, which phosphorylates AID at DNA site, and a complex of *cis* elements (reviewed in [30, 31]). Future comprehensive genome-wide screening of mutated genes and gene fusions will provide a more defined picture of the mechanisms regulating AID targeting.

## AID in inflammatory processes

It is generally accepted that expression of AID and class switch process occur in lymphoid tissue. Important novel aspect linking B-cell biology and inflammation is based on the discovery of GC-like structures outside of secondary lymphoid organs. Accumulating evidences demonstrate the existence of AID-positive ectopic follicular structures at sites of inflammation within different tissues, suggesting class switch recombination and somatic hypermutation events to take place locally. One of the pioneer work [32] has demonstrated that IgE-committed B cells do not necessarily need to migrate through the circulation to the nasal mucosa; detection of AID mRNA, multiple germline gene transcripts, and ε circle transcripts in the nasal mucosa of allergic subjects indicated that CSR occurs locally in allergic rhinitis. Later, local expression of AID and CRS to IgE was shown in the bronchial mucosa of atopic and non-atopic patients with asthma [33]. Additionally, the esophageal mucosa was proposed to be a site for initiation and development of humoral responses given the occurrence of AID expression, local immunoglobulin class switching to IgE, and IgE production in the esophageal mucosa of patients with eosinophilic esophagitis [34]. Indeed, the esophageal mucosa possesses a strong immunological capacity based on a diversity of resident immune cell types, in particular, B lymphocytes, T cells, mast cells, and dense eosinophilic infiltration as well as the presence of Th2 cytokines IL4 and IL13. The authors suggested that sensitisation and activation of mast cells involving local IgE may critically contribute to the pathogenesis of eosinophilic esophagitis. Furthermore, recent findings indicate that the presence of AID-positive ectopic lymphoid structures can be detected in chronically inflamed tissues in several autoimmune disorders [35]; in synovium of rheumatoid arthritis, the AID-positive follicular structures are directly implemented in promoting the production of pathogenic autoantibodies [36]. The data suggest that tissues under constant antigenic challenge (e.g., the intestinal, nasal and bronchial mucosa) support B-cell activation, AID expression, isotype switching, and Ig production. It is yet unclear what the crucial endogenous checkpoints are being necessary for mounting a limited, positively effective inflammatory response with participation of B lymphocytes and what the borderline is converting the physiological ectopic follicles to the pathophysiological autoantibody-producing structures.

## Multigene signature approach to delineate the role of AID-driven events under Th2 supervision

The establishment and validation of gene-, pathway-, or disease-relevant signatures provides tools for understanding the functional relevance of gene alterations in human diseases not only for basic research but also for therapeutic target proposal, diagnostic tools, and therapy response monitoring [37–40]. The implemented methodology to study gene expression profiles of modules with particular biological function(s) in the etiology of disease may vary: (1) in silico data-driven approaches using microarray data analysis offer the advantage of a transcriptome-wide screening procedure but often lack the sensitivity for genes expressed at low levels; (2) a knowledge-driven approach offers the detailed characterization of the input of one particular pathway while keeping the amount of genes limited at the beginning of the study. In this case, the composition of a core set of genes can be assembled based on the data mining (scientific literature, creation of gene interactive networks) with subsequent application of the designed multigene signature for real-time PCR-based gene profiling. Thereby, the important advantages are the high sensitivity and reproducibility allowing quantitative profiling even of low-copy genes that are below the detection limits of microarray platforms.

**Fig. 2:**
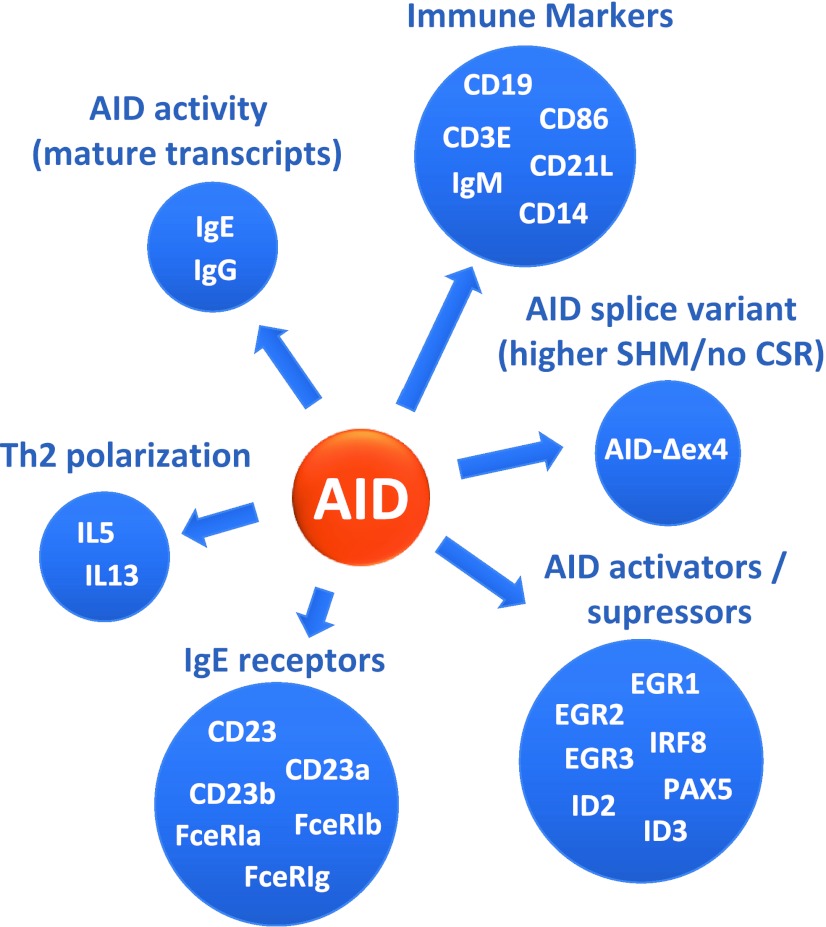
Th2-type AID-associated multigene signature. The composition of the signature created around AID as a node gene allows to assess (1) AID expression and AID activity as proven by the class switch recombination-based formation of IgG and IgE mature transcripts; (2) the presence of tissue infiltrating immune cells such as B cells, T cells, monocytes, and follicular dendritic cells being indicative for various stages of lymphoid organization; (3) the expression pattern of low- and high-affinity IgE receptors mediating numerous IgE-related immune responses; and Th2 polarization [21]

We have used recently the second approach to create the multigene signature using AID (NM_020661) as a node gene [21]. The self-designed 25-gene “AID signature” included the full-length AID-FL and the alternative AID splice variant AID-Δex4; activators and suppressors in AID regulation; immune cell markers; Th2 cytokines; low- and high-affinity IgE receptors; and IgM, IgG, IgE mature transcripts (Fig. 2). This signature was evaluated in a disease model of benign, chronically inflamed tissue, namely in nasal polyposis. In this study, we have shown, to our knowledge, for the first time that AID is expressed within the nasal polyp tissue. Comparison of gene expression patterns for chronic rhinosinusitis with and without nasal polyps confirmed an AID-specific gene signature for the disease state with nasal polyps. The data suggested that the local environment created within nasal polyp tissue may trigger formation of ectopic follicular structures with AID expression/activity and consequently initiate CSR. This was in turn proven by the detection of strongly elevated IgG and, particularly, IgE mature transcripts, fully supported by immunostaining. Notably, AID mRNA was found to have a strong positive correlation with Th2 players IL13 and IL5. Furthermore, arrangement of datasets for each specimen across all genes was able to provide the gene expression pattern characteristic for each individual sample and therefore being patient orientated. Thus, using a multigene signature covering one particular disease-associated module with AID as the key gene, we further explored associations between AID and other molecules involved in the etiology of human inflammation-driven disease such as nasal polyposis: in addition to the previously highlighted biomarkers/targets such as IgE and IL5, novel players were suggested including among others IL13 and CD23 [21].

## Multifaceted AID: functional link to tumorigenesis

Last years findings clearly indicate that, in addition to diversifying the immune repertoire, AID can also target non-Ig genes. Thus, an aberrant hypermutation activity targeted multiple loci, including the proto-oncogenes PIM1, MYC, RhoH/TTF (ARHH), and PAX5, in more than 50 % of diffuse large-cell lymphomas (DLCLs), which are tumors derived from germinal centers [41]; human BCL6 gene was shown to be also hypermutated in peripheral blood memory B cells and tonsils [42]. Intriguingly, even the T-cell receptor in AID-transgenic mice [43] and a GFP plasmid reporter gene in AID-transgenic fibroblasts [5] were shown to hypermutate. Wang et al. demonstrated that hypermutation requires no Ig gene sequences; instead, AID (and possibly other trans-acting hypermutation factors) may function as general, genome-wide mutator [44]. Thus, by mutating multiple genes, and possibly by favouring chromosomal translocations, AID-driven aberrant, non-physiological hypermutation likely represents one of the major contributors to lymphomagenesis. In support, AID acts as mutator in the Philadelphia chromosome plus (Ph^+^) BCR-ABL1-transformed acute lymphoblastic leukemia (ALL) cells [45].

**Fig. 3:**
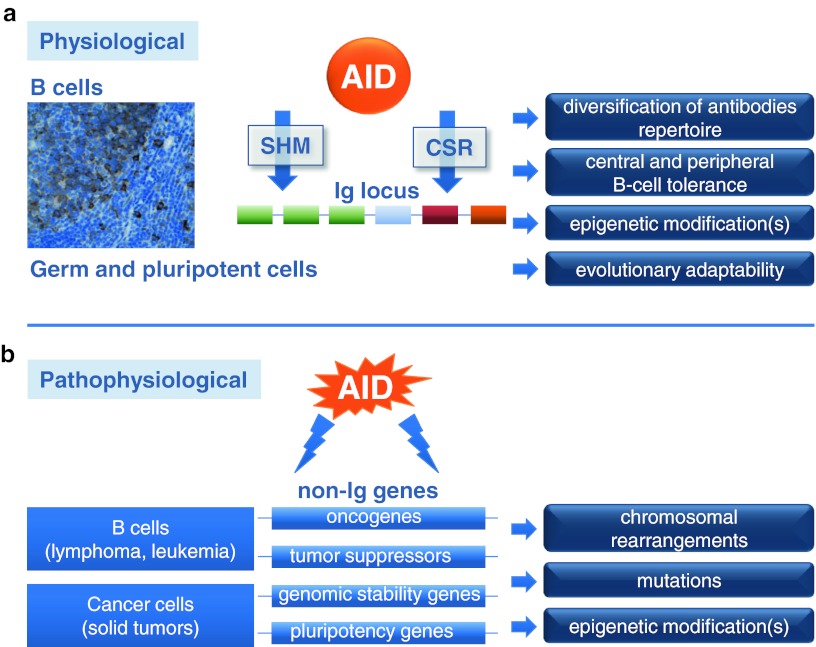
AID from immunity to cancer. **a** Somatic hypermutation (SHM) and class switch recombination (CSR) of B-cell immunoglobulin (Ig) genes are AID driven. Recently, a new role for AID in active DNA demethylation and reprogramming of mammalian somatic cells toward pluripotency has been identified [65]. Furthermore, requirement for AID in several crucial steps of B-cell terminal differentiation and for the establishment of both central and peripheral B-cell tolerance was proposed [11]. Accumulating evidence suggests that the AID/APOBEC family members could be important drivers of evolutionary adaptability [12]. **b** Under pathophysiological circumstances, AID may target non-Ig genes, including cancer-related genes as oncogenes, tumor suppressor genes, genomic stability genes, and pluripotency genes [42, 45, 47, 51, 66]. Picture insert; AID-positive GCs within tonsil tissue. To detect AID, mouse IgG1-kappa antibodies, clone ZA001 (Invitrogen) and DAKO EnVision+, Peroxidase system (DAKO, Glostrup, Denmark) was used as previously described [21]

If to look deeply and make approximation to other types of tumors, the link between functionality of AID and malignancies becomes more transparent and logical: cancer cells acquire tumor-specific DNA alterations, including multiple somatic mutations of tumor-promoting genes and/or recurrent chromosomal translocations and their corresponding gene fusions at the precise timing during cellular development. A total of 358 gene fusions involving 337 different genes have been identified (reviewed in [46]). An increasing number of gene fusions are being recognized as important diagnostic and prognostic parameters in distinct malignant disorders. Then, abnormal AID expression and functionality in non-B cells could have a strong contribution to human malignancy in general, including solid tumors (Fig. 3); probably, not only the level of overexpression but also constitutive versus transient manner is important. Indeed, accumulating evidences suggest that an aberrant AID activity in epithelial tissues may provide the critical link between inflammation, somatic mutations, and cancer development [47]. Given the analogy to the mechanisms of AID activation in B cells, Th2 direction might play an essential role. Thus, the potential contribution of AID to the development of gastric cancers was proposed [48]. Constitutive AID expression was detected in 6 out of 6 malignant epithelial cells of breast cancer origin and the cell line derived from uterine cervix, suggesting a potential for inducible aberrant mutational activity [49, 50]. AID expression leading to increased mutation rate of TP53 was recently shown in human lung cancer cell lines [51]. The proinflammatory cytokine-induced production of AID was proposed to link bile duct inflammation to cholangiocarcinogenesis [52]. AID expression was shown to be triggered by TNF-α or IL-1β in human hepatocytes [53]. Strong evidence indicates that AID might contribute to the development of colitis-associated and inflammatory bowel disease (IBD)-associated colorectal cancers by linking colonic inflammation to an enhanced genetic susceptibility to oncogenic mutagenesis [54–56]. Importantly, aberrant AID expression in human colonic epithelial cells was induced by TNF-α via NFκB-dependent pathway and by Th2-driven cytokines IL4 and IL13 in a STAT6-dependent manner. Both cytokines are critical mediators of mucosal inflammation; accordingly, IL13 secreted by natural killer T cells is an important pathologic factor for ulcerative colitis [57]. Furthermore, it has been shown that several oncogenic viruses can induce AID expression. Hepatitis C virus (HCV)—one of the leading causes of hepatocellular carcinoma—strongly triggers AID expression in hepatocytes in collaboration with proinflammatory cytokines [53], and ectopic AID expression is observed in human liver specimens with chronic hepatic inflammation caused by HCV infection [58]. Another intriguing evidence linking oncogenic infection with ectopic AID expression is attributed to Epstein–Barr virus (EBV); LMP2A, EBV latent membrane protein 2A, is able to strengthen B-cell receptor (BCR)-mediated signaling pathways leading to the AID activation [59]. However, thus far such mechanism was demonstrated only for B cells, emphasizing a potential role for the development of the EBV-positive, GC-associated lymphomas [60]. Next potential link of AID to tumorigenesis is based on the fact that the Aid and Apobec1 genes are located in a cluster of pluripotency genes including Nanog and Stella and are co-expressed with these genes in oocytes, embryonic germ cells, and embryonic stem cells. These data suggest that AID and perhaps some of other APOBEC family members may have roles in epigenetic reprogramming and cell plasticity, contributing to tumor etiology [61]. An important functional link was identified between estrogen and AID: estrogen directly activates expression of AID [62] revealing yet another connection between AID regulation and cancer, particularly the gender aspect for some cancers. Therefore, in tissues where estrogen levels are continuously high, AID-driven aberrations may accumulate with time, which in turn might contribute to the development and/or progression of estrogen-dependent tumor types. In analogy, female gender bias in allergy associated with endogenous and exogenous estrogens [63] may be caused by the AID/estrogen axis.

Although the major focus of the data summary in this sub-chapter of the article was given to the AID-positive tumor cells, tumor-associated AID-positive B cell should not be underestimated. Hypothetically, identification of AID-positive B-cell infiltrates indicates local maturation and priming for class switching. Moreover, infiltrating B cells might modulate the malignant potential of tumor cells via the production of certain chemokines or cytokines. Therefore, the biological responses initiated within AID-positive ectopic follicular structures in the tumor or tumor-stroma microenvironment may influence the disease pathogenesis, progression and/or disease resolution.

Increasing knowledge about AID, understanding the AID-associated responses, both in B cells and tumor cells, might allow stratifying the prognosis of various cancer types and considering whether targeting of AID is beneficial for AID-positive tumors as suggested recently for plasmocytoma [64] and colitis-associated colon carcinogenesis [55].
